# Users’ Perceptions of Access to and Quality of Unified Health System Services in Brazil: A Cross-Sectional Study and Implications to Healthcare Management Challenges

**DOI:** 10.3390/ijerph21060721

**Published:** 2024-05-31

**Authors:** Jhoyce S. Souza, Edna A. Reis, Brian Godman, Stephen M. Campbell, Johanna C. Meyer, Luann W. P. Sena, Isabella P. D. Godói

**Affiliations:** 1 Institute of Pharmaceutical Sciences, Federal University of Rio de Janeiro, Avenida Aluízio da Silva Gomes 50—Granja dos Cavaleiros, Macaé 27930-560, RJ, Brazil; jhoyce.tcc.ufrj@gmail.com; 2 Department of Statistics, Exact Sciences Institute, Federal University of Minas Gerais, Campus Pampulha, Avenida Antônio Carlos 6627, Belo Horizonte 31270-901, MG, Brazil; 3 Strathclyde Institute of Pharmacy and Biomedical Sciences, University of Strathclyde, Glasgow G4 0RE, UK; 4 Department of Public Health and Pharmacy Management, School of Pharmacy, Sefako Makgatho Health Sciences University, Ga-Rankuwa, Pretoria 0208, South Africa; stephen.campbell@smu.ac.za (S.M.C.); hannelie.meyer@smu.ac.za (J.C.M.); 5 School of Health Sciences, University of Manchester, Manchester M13 9PL, UK; 6 College of Public Health, Institute of Health and Biological Studies, Federal University of South and Southeast of Pará, Avenida dos Ipês, s/n, Cidade Universitária, Loteamento Cidade Jardim, Marabá 68508-970, PA, Brazil; luannsena@unifesspa.edu.br; 7 Health Technology Assessment Center—Management, Economics, Health Education and Pharmaceutical Services (GEESFAR/NATS/UFRJ), Federal University of Rio de Janeiro, Avenida Aluízio da Silva Gomes 50—Granja dos Cavaleiros, Macaé 27930-560, RJ, Brazil

**Keywords:** Brazil, access, quality, health services, experiences, users, public health, management

## Abstract

Evaluating the access to and quality of healthcare services from the users’ perspective is an important assessment process to identify priorities. This study assessed the profile of health service usage and the views of the Unified Health System (SUS) users about the access to and quality of SUS public health services. A cross-sectional study was conducted with participants from the Coastal Lowlands Region of the Rio de Janeiro State/Brazil, between August and November 2023. The association between categorical variables was analyzed using the Pearson Chi-Square test, using R software 4.3. A total of 200 individuals were interviewed using a 66-question survey instrument. Participants who reported using SUS services more frequently rated this system as essential (*p*-value = 0.031). However, overall, 64% of participants rated the quality of care to be *very bad/bad and 34.9% rated access as very bad/bad. Access was considered poor by respondents who used public services rarely or sometimes* (*p*-value = 0.002). In terms of accessing SUS services consultations provided by specialists (e.g., neurologists), these were available only in another municipality (*p*-value = 0.001). Many participants were SUS dependent for health services, and gaps and weaknesses were observed regarding users‘ perspectives of the access to and quality of SUS health care. Policymakers should prioritize evaluations and dialogue with the community to make SUS services responsive and to optimize value-for-money in health service planning.

## 1. Introduction

In Brazil, free access to health services for the population in the country became a right of citizenship from the Federal Constitution of 1988, in article 196 [1]. The regulation of the Brazilian public health system occurred with the Laws n° 8080 [2] and n° 8142 [3] of 1990, which established a universal health system based on key principles including completeness, decentralization, and hierarchical and social participation [2,3,4,5]. According to Castro and collaborators (2019), the trajectory of the development and expansion of the Unified Health System (SUS) has faced many challenges in fulfilling its constitutional mandate of providing ‘health for all’ in a highly unequal country with relatively low resources and highlighted the importance of community participation [6,7].

The SUS organizes and provides health services and actions at different levels in Brazil, including primary, secondary, and tertiary care [6]. Evidence suggests that primary care-led health care systems increase the responsiveness and capacity to address issues locally [8,9] and enable greater capacity to address the World Health Organization (WHO) Sustainable Development Goals (SDGs) and Universal Health Coverage (UHC). The joint WHO/World Bank/OECD 2018 report stated: “accessible and high-quality primary care should be the bedrock for all other services” [10] and advocated a focus on seven domains of quality of care. These were effectiveness, safety, people-centeredness, timeliness, integration of care, equity, and efficiency. According to the Ministry of Health in Brazil, the majority of health demands can be addressed in primary care [2], which emphasizes its importance to health systems as the main entry point for patients to the SUS in Brazil [11,12,13].

Over the past few years, many projects and strategies have been promoted by the Ministry of Health in Brazil aimed at the primary health care level, including the National Immunization Program (PNI, in Portuguese), which has guaranteed the provision of vaccines to the population since its establishment in 1973 [14,15]. The PNI, as part of the SUS, provides fifteen vaccines to children, nine to adolescents, and five to adults and the elderly, free of charge, from Basic Health Units and Family Health Strategies, protecting the population against more than 20 diseases. Morbidity and mortality have been reduced due to this public health strategy, with a focus on prevention, and key program of the SUS, as it significantly contributed to the control of various vaccine-preventable diseases [14]. These include the eradication of smallpox in 1973, the elimination of rubella and congenital rubella in 2015, measles in 2016, and neonatal tetanus in 2020 [15].

During the COVID-19 pandemic, the PNI facilitated collective immunization against SARS-CoV-2, following the introduction of effective vaccines across different age groups [14,16]. The Brazilian government financed the purchase of vaccines. Up until March 2024, a total of 553,562,962 doses have been administered in the country against SARS-CoV-2 since the start of the vaccinations [16]. The COVID-19 immunization services were delivered by Primary Health Care and reinforced the PNI as one of the most important health programs in Brazil, included in the SUS.

The SUS provides a network of services to over 203 million inhabitants, distributed among over 5500 municipalities (26 States and the Federal District) throughout the country, of which approximately 70% have populations of between 10 and 20 thousand inhabitants [17]. The importance of local collaboration and partnership enables the provision of responsive health services to their respective populations, given the numerous limitations and challenges in logistics and infrastructure, as well as the cultural and socioeconomic issues faced in health care delivery by each locality [6,7]. Consequently, it is relevant to conduct studies involving local, regional, or national interventions, in order to better evaluate the perception of users in terms of access to and quality of SUS health care in Brazil.

Alongside SUS services, the Brazilian private market for health insurance is regulated by the National Regulatory Agency for Private Health Insurance and Plans, which works on behalf of the Ministry of Health [18]. Private health insurance can either be purchased individually or obtained as a work benefit, depending on the employer. Individuals who decide to purchase private health insurance may still access public health care [19]. According to National Regulatory Agency for Private Health Insurance and Plans, 51,035,365 individuals have private health insurance (March 2024) in the country, approximately 25% of the population [20].

The timely access to health care services is a crucial component of, and a prerequisite for, delivering quality of care [21,22]. There have been some studies published regarding Brazil in relation to the management of public health services at the federal, state, and municipal levels [23,24,25]; the quality of health services [12,26,27,28,29]; and about pharmaceutical public services [30,31,32,33].

The concept of access is broad and requires a multidimensional understanding with political, socioeconomic, technical, and organizational aspects, with the goal of enabling users to use health services to meet their needs [34]. It is important to differentiate between the terms access, accessibility, and availability and to differentiate between timely access to health services and physical access to services. Indeed, access involves key aspects such as geographic dimensions, economy, culture, and services offered [35]. Given that Brazil is a country with many local and regional particularities inside and outside the health scenario, it is relevant to evaluate the contexts of the health service in terms of the access and quality of services offered from the SUS user’s perspective in different localities.

According to the WHO’s 2016 publication “*Global strategy on human resources for health: Workforce 2030*”, in the Western Pacific Regional Office, the UHC is part of a broader concept of universal access to health care, highlighting the need to achieve improvements in the access, acceptability, and quality of health services [36]. Quality primary care services improve population and individual health outcomes, contribute to the reduction in public health costs, achieve a greater efficiency of care, and enable the identification of quality deficits in health care services such as waiting times or unavailability of health professionals or services [37,38,39,40]. Identifying and improving deficiencies will be a key focus for future programs.

The present study aimed to ascertain the perceptions and experiences of individuals from the Coastal Lowlands, in the Rio de Janeiro State, regarding the access to and quality of services offered by the SUS, as well the profile of use of health services in this region in Brazil. Understanding SUS users’ perceptions and needs is essential for the monitoring and assessment of public health systems and for assisting with the future planning and management of health services in this region.

## 2. Methods

### 2.1. Study Design and Setting

A cross-sectional study was conducted to assess the views of individuals in the Coastal Lowlands of Rio de Janeiro State, regarding the access to and quality of SUS health services. This study was conducted in two steps—Step One: descriptive questions and analysis including all participants (n = 200) related to general aspects of health services and the sample characteristics (e.g., gender, age, education, and family income); Step Two: users’ perceptions regarding the access to and the quality of the public health system/SUS.

**Figure 1:**
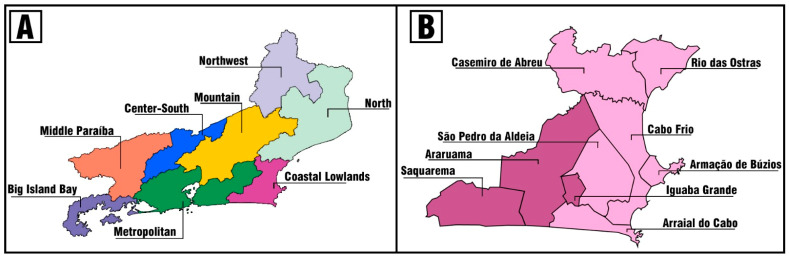
(**A**) Rio de Janeiro State in Brazil. (**B**) Coastal Lowlands Region of Rio de Janeiro State. Note: the municipalities involved in this study were highlighted in a light pink color (B).

Within Brazil, the Rio de Janeiro State is in the southeast region of the country, with approximately 16 million inhabitants [41], divided into eight regions, i.e., the Metropolitan, Medio Paraíba, Central-South Fluminense, Mountain, Coastal Lowlands, Fluminense North, Fluminense Northwest, and Big Island Bay, as presented in Figure 1A [42]. The Coastal Lowlands is one of the main regions of the State, containing nine municipalities, including Cabo Frio and Rio das Ostras, as illustrated in Figure 1B [43]. The population of the region of the study has grown significantly in recent years and has undergone many socioeconomic transformations, including direct and indirect resources derived from oil exploration and tourism [44]. These changes have contributed to an increase in the demands on health services, without an understanding of its residents’ views about the public health care services offered. To the best of our knowledge, no study has evaluated user experience of the access to and quality of health services in this region.

This study included the six main municipalities of this region in terms of demographic and socioeconomic characteristics such as Rio das Ostras, Cabo Frio, Casimiro de Abreu, São Pedro da Aldeia, Armação dos Búzios, and Arraial do Cabo, located between 135 and 170 km from the state capital (Rio de Janeiro) [45]. The municipality of Cabo Frio has 222,161 inhabitants and a GDP *per capita* of BRL 52,801.54 (USD 10,475.69) [46], while Rio das Ostras has 156,491 inhabitants and a GDP *per capita* of BRL 56,096.82 (USD 11.126,34) [47].

### 2.2. Survey Instrument and Pilot Study

The questionnaire was prepared with the participation of students from the UFRJ-Macaé Pharmacy Course IPDG. The majority of questions included in the questionnaire were obtained from previous projects of the Brazilian Ministry of Health [48,49]. Overall, an interviewer-administered questionnaire (Supplementary Materials S1) was created from publications of the Ministry of Health, such as the National Survey on the Access, Utilization and Promotion of Rational Use of Medicines (PNAUM), and the National Program for Improving Access and Quality of Primary Care (PMAQ) [48,49]. The instrument contained 66 questions, organized into the following four sections: (A) Socioeconomic and Use of Health Services Profile; (B) Clinical Condition; (C) Medication Use; and (D) Perceptions and Use of Public Health Services. It should be reinforced that in the last section (D), only the participants who reported use of SUS services answered (Step Two: Assess the users’ perceptions regarding access to and the quality of SUS services) and the previous sections (A, B, and C) included all participants (n = 200) (Step One—Descriptive Analyses).

To enhance the robustness of the questionnaire, it was pre-tested with 30 individuals from the municipality of Macaé (Northern Fluminense Region) near to the Federal University of Rio de Janeiro (UFRJ-Macaé). Comments were included in the final questionnaire for the main study. The pre-testing confirmed that no questions needed to be changed.

### 2.3. Data Collection and Inclusion Criteria

Data collection was undertaken between August and November 2023 in the Coastal Lowlands Region, involving 6 municipalities, as described above. The sample size calculation followed the proportion of the respective regional population. This resulted in a final minimum sample of 200 participants, which ensured a maximum margin of error of 7% in the estimation of global percentages.

Individuals aged 18 years or older (legal majority in Brazil) were recruited by convenience sampling, allowing the researchers to obtain a range of attitudes and opinions [50]. Participants who declared that they never used SUS services were asked questions in Step One (sections A, B, and C). Participants that reported using SUS services were asked questions in both Step One (Sections A, B, and C) and Step Two (section D), which related to users’ perceptions about the access to and quality of SUS services.

In addition, individuals who reported only purchasing medicines in private pharmacies were also excluded from Step Two, which involved questions regarding pharmaceutical services in the public health system.

Data collection was conducted by five undergraduate students from the School of Pharmaceutical Sciences at the Federal University of Rio de Janeiro/Macaé who had been trained by one of the investigators (IPDG). The survey questions were administered in an interview, which was conducted in Portuguese in public spaces including public markets, squares, and avenues. Participants were invited to take part in the research on a voluntary basis and the objectives of the research were explained. Participants who agreed to participate in the research were asked to read and sign two copies of a consent form, one for the participant and one for the researcher.

### 2.4. Data Analysis

Analyses were conducted using the Microsoft Excel 2019 and R software version 4.3.0. Step One consisted of a descriptive analysis of responses from all participants (n = 200) relating to the use of SUS public health services including primary care, pharmacy, and specialist services and the sample characteristics, e.g., gender, age, education, and family income. Step Two focused on assessing users’ perceptions of the access to and the quality of the SUS public health system excluding individuals who declared never using SUS services (primary care or pharmacy or specialist services) in Step One. In Step Two, questions related to use of pharmacies and included users’ experiences of receiving guidance on the use of medicines and the role and presence of a pharmacist.

In addition, we assessed users’ experiences of obtaining and using both over-the-counter (OTC) and prescribed medicines, as well as their adherence with taking prescribed medicines. Additionally, we evaluated their understanding of medicine information leaflets, including guidance on taking antibiotics and advice on the concomitant use of alcohol, as well as polypharmacy.

Categorical variables were described by absolute and relative frequencies. The association between categorical variables was analyzed using the Pearson Chi-Square test and was considered statistically significant when the *p*-value was <0.05.

In addition, the conversion value provided by the Central Bank of Brazil (2023: USD 1, BRL 5.04) was adopted [51].

### 2.5. Ethical Aspects

This study was approved by the Research Ethics Committee of the Federal University of Rio de Janeiro/Macaé Campus (CAAE: 68864623.6.0000.5699).

## 3. Results

### 3.1. Population Characteristics

The data collection involved 200 participants from different locations and socioeconomic characteristics in the six localities of the Coastal Lowlands Region/Rio de Janeiro State. In Step One, 67% of respondents were female and 97.5% of individuals reported using SUS services (n = 195). The average age of the interviewees was 44 years (SD ± 13). In total, 64% of the respondents purchased their medicines privately only and 43.1% reported having purchased a medical prescription. Further details on the characteristics of the respondents are shown in Table 1.

Hypertension, anxiety, depression, diabetes mellitus, arthritis, and respiratory diseases were the most common self-reported conditions among participants, with 38% of the sample reporting two or more diseases and 23% of participants reporting no clinical conditions. The clinical profile of participants is shown in Table 2.

**Figure 2:**
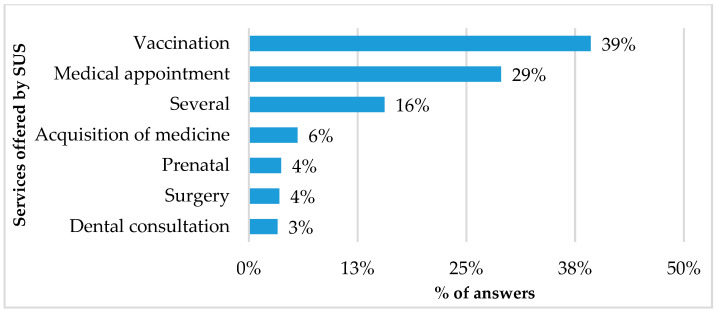
Profile of SUS services used by respondents (n = 200). Note: respondents could record more than one service.

Among the individuals who declared using SUS services (n = 195), 128 (64%) reported purchasing medicines in private pharmacies, while 67 (36%) purchased medicines in public pharmacies. Respondents reported using SUS services mostly for vaccination (39%) and medical appointments (29%). See Figure 2 for further details.

**Figure 3:**
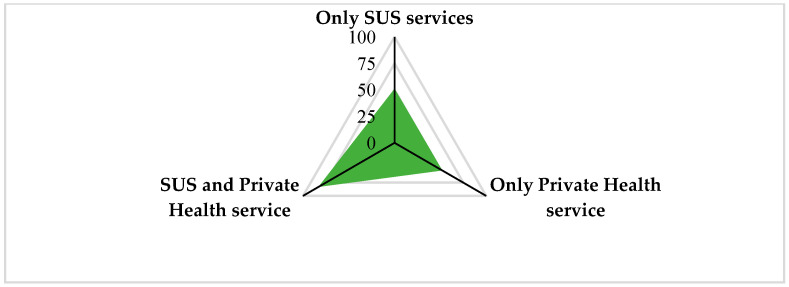
Profile of medical consultations used by the respondents (n = 195). Note: a total of five participants did not want to respond to this question.

Overall, 42.1% of respondents declared using both the SUS and the private care (health plans or individually) for medical consultations, 26.4% used only private care, 26.0% used only SUS services, and 5.5% did not want to respond to this question (see Figure 3).

**Figure 4:**
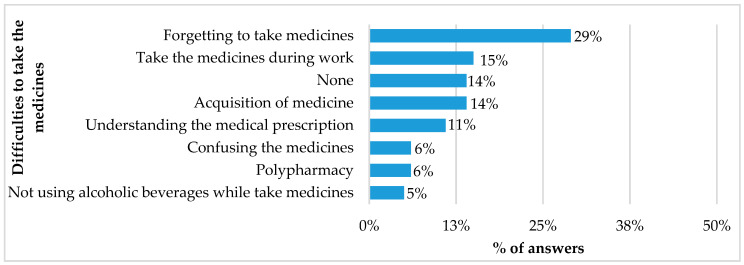
Difficulties reported by respondents associated with the use of medicines (n = 200). Note: respondents could record more than one service.

The majority (87.7%) of the respondents reported using OTC medicines without a prescription and also reported “using the medication as it is already at home”. However, several difficulties were faced by the respondents regarding the use of medicines, including forgetting to take medicines (29%) and obtaining medicines from SUS services (14%), as presented in Figure 4.

Participants identified key priorities for improvement in SUS services, with 70.6% of respondents reporting issues related to patient safety, functionality, and comfort within SUS services. In addition, 12.1% of participants reported that there should be an increase in the number of SUS units to enhance accessibility, while 10.7% indicated that there should be an improvement in the ease of obtaining medications from the SUS public pharmacies.

### 3.2. Step Two: SUS Users’ Perceptions Regarding Access to and Quality of Health Services

Of the 200 participants in the sample, as mentioned, 195 (97.5%) reported using the Brazilian SUS public health system. Participants who used public health services more frequently (*always* and/or *often*: n = 195) placed a greater importance on the SUS as being indispensable and essential to the population. Those who use them *sometimes* and/or *rarely* reported viewing the SUS as a complementary health system (*p*-value = 0.031), as presented in Table 3.

In general, access was considered poor for respondents who used public services *rarely* or *sometimes* (*p*-value = 0.002). Additionally, in a correlation analysis between the variables of the quality of SUS services and the frequency of use of these services, those who use public health services more frequently tended to consider the quality of care as neither good nor bad (*p*-value = 0.000).

When questioned about the presence in the pharmacy of a qualified pharmacist at the time of medication acquisition, pharmacists were reported as generally not being present by users of SUS public pharmacies, with 60% declaring never having spoken with a pharmacist. Those who acquired medicines through public and private services reported the presence of a pharmacist *always* (18.8%), *frequently* (18.8%), and *sometimes* (43.8%).

Individuals who only acquired medicines through SUS pharmacies considered the role and/or contribution of the pharmacist as being indifferent to the guidance process for the use of the medication, and those who acquired medicines through both services (public and private) considered a pharmacist as *indispensable* and *essential* (*p*-value = 0.0005), as shown in Table 4.

In terms of accessing consultations with specialist SUS services, e.g., pediatric neurologists and cardiologists, consultations were available only in another municipality (*p*-value = 0.001). No statistically significant results (*p*-value > 0.05) were found in relation to the participants’ views of infrastructure, workforce, and services, as shown in Table 5.

## 4. Discussion

We believe this is the first study to evaluate users’ experiences of access to and quality of the public health services in the southeast of Brazil. The majority of participants used the public health system (97.5%) and the results demonstrated that SUS services such as vaccinations are used regularly by the Brazilian population. Strong primary health care is essential for responsive health services to be offered for the community [8,9]. However, these results highlight major deficiencies from the perspectives of users of SUS services in the structure, processes, informational, clinical and administrative/organizational activities of the SUS in Brazil [12,53].

Overall, only 23% of participants rated the quality of care to be *very good* or *good* and only 18.9% rated access as *very good* or *good*. Participants that used SUS services *frequently* and *sometimes*, 61.8% and 71.1%, respectively, reported the quality of the SUS public health services as *neither good nor bad*. Moreover, access to SUS services were rated as *neither good nor bad* by 86.2% of participants who used SUS services frequently and 77.7% of those who used SUS services *sometimes* (*p*-value = 0.002). These data also showed that some pregnant women reported that prenatal care services are unsatisfactory in some municipalities of this region. Moreover, only 26% of participants in this study reported always being able to obtain a desired medicine at SUS public pharmacies, including both OTC and prescription medicines. Boing and colleagues (2022) also found that many individuals were unable to obtain medication through the SUS, which was associated with inefficient access to SUS services [54]. These results have, therefore, further demonstrated concerns by users of SUS services with the access to and quality of SUS healthcare services.

Almost half (47.5%) of the participants in this study reported having private health insurance, which is higher than the national average (∼25%) [20], although those who had private health plans or health insurance also reported using some SUS services. The number of individuals who are able to use a mix of public and private health services resonates with previous research [53]. The higher frequency of individuals who have private health insurance in this sample must be seen in the context of the deficits in the access to and the quality of care in SUS services found in this study in these municipalities.

The Coastal Lowlands Region is a municipality with less than 100 thousand inhabitants and an infrastructure that is insufficient to meet some health demands, e.g., maternity and specialist consultation, as well as procedures such as surgeries and transplants [43]. Participants in this study reported not being able to access consultations with specialists in their municipality of residence. Regionalization is a principle that underpinned the construction of the SUS system to promote and enable strategies for access to public services for the population. Where people reside in municipalities that are “lacking in infrastructure”, contingencies exist to provide free transport to consultations with specialists in other municipalities. According to Carvalho and collaborators (2017), the decentralized regionalization process has been instrumental in ensuring access to SUS services [55].

In the pharmacy context, many municipalities in the Rio de Janeiro State do not provide some services and activities such as the Family Health Strategy and Basic Health Units. Moreover, in public SUS pharmacies, medication dispensing is mainly performed by another member of staff other than a pharmacist, which contextualizes our finding that a considerable percentage of participants had never seen a pharmacist the SUS public pharmacies (60%). Peixoto et al. (2022) [56] and Torrês et al. (2024) [57] have emphasized that the presence of a pharmacist increases and optimizes medication dispensing and the delivery of technical knowledge about medication use to patients, which was valued and recognized in our sample, especially by interviewees who use private pharmacies (value-*p* = 0.0005) The presence of a pharmacist also promotes the rational use of medicines [56,57].

The study aimed to identify the experiences and views of the users of SUS services in a socioeconomically deprived region of Rio de Janeiro. However, some limitations should be noted including the participation of only six out of nine municipalities in the Coastal Lowlands Region, which have the most relevant socioeconomic impact. However, as presented in Table 1, the characteristics of the sample were similar to those of the Brazilian population as a whole [58]. The convenience sample used may not be generalizable to the wider population of Brazil. However, the convenience sample approach used in this study facilitated us to obtain a range of attitudes and opinions [50]. Despite these limitations, we believe the study has succeeded in its aim of identifying SUS service users’ views on the access to and the quality of SUS health services in the Coastal Lowlands Region of Rio de Janeiro State.

## 5. Conclusions

This is a unique study focusing on one of the main regions of an important Brazilian State, providing evidence of users’ perceptions of public SUS services. Overall, only 23% of participants rated the quality of care to be *very good* or *good* and only 18.9% rated access as *very good* or *good*, with more than 60% of SUS users assessing the access to and quality of SUS services as neither good nor bad. These findings highlight important priorities for improvement by SUS users for consideration by policy-makers in planning and delivering micro–meso–macro reforms to improve future access to and quality of responsive SUS services and universal health coverage.

Many people in Brazil are SUS dependent for healthcare. The SUS has been essential in promoting health and meeting the health needs of many of the Brazilian population, ensuring access to health services, promoting disease prevention, and contributing to the improvement of the quality of life of millions of Brazilians. Its important role is intrinsically linked to the improvement of the health of the population, promoting a primary care-led, more efficient, accessible, and patient-centered health system. These findings and their implications can help towards this goal.

## Figures and Tables

**Table 1:** Characteristics of the respondents (n = 200).

**Variable**	**n**	(%) *
Female	134	67.0%
Male	66	33.0%
***Age Profile (years old)***		
18–25	21	10.5%
26–45	103	51.5%
46–60	54	27.0%
More than 60	22	11.0%
***Race/skin color***		
White	73	36.5%
Black	30	15.0%
Brown	94	47.0%
Other	3	1.5%
***Education level***		
Never attended school	2	1.0%
Incomplete elementary education	21	10.5%
Completed elementary school	14	7.0%
Incomplete high school	10	5.0%
Completed high school	54	27.0%
Incomplete college	36	18.0%
Completed college or more	63	31.5%
Family income * (number of times the minimum wage) **		
Up to 1	17	8.5%
1–2	46	23.0%
2–3	42	21.0%
3–5	51	25.5%
5–10	27	13.5%
10–20	3	1.5%
>20	2	1.0%
***Use of SUS services—Yes***	195	97.5%
***Has a private health plan—Yes***	95	47.5%

Notes: * Family income: Some respondents did not answer these questions (“don’t know/don’t want to answer”); ** Minimum wage in 2023: BRL 1320.00 [52].

**Table 2:** Clinical profile of participants that reported their illnesses (n = 154).

Clinical Condition	%
Other respiratory diseases	26.2%
Anxiety/Depression	13.8%
Hypertension	12.4%
Other diseases	12.0%
Chronic Obstructive Pulmonary Disease	11.3%
Dyslipidemia	10.5%
Joint Disorders	7.7%
Diabetes Mellitus	3.1%
Other Cardiovascular problems	3.0%

Note: in total, 46 (23%) participants reporting no clinical conditions.

**Table 3:** Perceptions of SUS users regarding the relevance, access to, and quality of the public health services (n = 195).

	***Relevance of SUS* n (%)**							
***Frequency***	**Indispensable/Essential**			**Complementary**		**Indifferent**		***p*-Value**
*Always*	27 (81.8%)			3 (9.1%)		3 (9.1%)		0.031
*Frequently*	37 (100.0%)			0 (0.00%)		0 (0.00%)		
*Sometimes*	74 (88.1%)			6 (7.1%)		4 (4.8%)		
*Rarely*	34 (82.9%)			7 (17.1%)		0 (0.00%)		
*ALL*	172 (88.2%)			16 (8.2%)		7 (3.6%)		
	**Access to SUS services n (%)**							
***Frequency***	Very good	Good	Neither good nor bad		Bad		Very bad	*p*-value
*Always*	2 (5.7%)	12 (34.3%)	14 (40.0%)		5 (14.3%)		2 (5.7%)	0.002
*Frequently*	0 (0.00%)	5 (13.8%)	21 (55.6%)		11 (30.6%)		0 (0.00%)	
*Sometimes*	1 (1.2%)	10 (12.8%)	39 (47.5%)		23 (29.5%)		7 (9.0%)	
*Rarely*	3 (7.3%)	3 (7.3%)	15 (34.15%)		12 (29.3%)		9 (21.9%)	
*ALL*	6 (3.1%)	30 (15.8%)	90 (44.7%)		51 (26.7%)		18 (9.7%)	
	**Quality of SUS services n (%)**							
***Frequency***	Very good	Good	Neither good nor bad		Bad		Very bad	*p*-value
*Always*	4 (11.8%)	8 (23.5%)	14 (38.3%)		8 (23.5%)		1 (2.9%)	0.000
*Frequently*	1 (2.7%)	5 (13.5%)	17 (43.3%)		15 (40.5%)		0 (0.0%)	
*Sometimes*	0 (0.00%)	14 (16.9%)	51 (60.2%)		14 (16.9%)		5 (6.0%)	
*Rarely*	4 (10.8%)	8 (21.6%)	10 (24.4%)		9 (24.3%)		7 (18.9%)	
*ALL*	9 (4.7%)	35 (18.3%)	92 (46.1%)		46 (24.1%)		13 (6.8%)	

**Table 4:** Perception of SUS users applied to pharmacists and their services (n = 195).

	***Presence of the Pharmacist in the Pharmacy* n (%)**							
***Acquisition of*** ***Medicines***	**Always**	**Often**	**Sometimes**		**Rarely**		**Never**	***p*-Value**
*Public Pharmacy*	1 (10.0%)	2 (20.0%)	1 (10.0%)		0 (0.0%)		6 (60.0%)	0.001
*Public and Private*	6 (18.8%)	6 (18.8%)	14 (43.8%)		5 (15.6%)		1 (3.1%)	
*ALL*	7 (16.7%)	8 (19.1%)	15 (35.7%)		5 (11.8%)		7 (16.7%)	
	**Role of the Pharmacist in patient guidance n (%)**							
***Acquisition of*** ***medicines***	**Indispensable/Essential**			**Indifferent**		**Unnecessary**		***p*-value**
*Public Pharmacy*	2 (22.2%)			6 (66.7%)		1 (11.1%)		0.0005
*Public and Private*	42 (97.7%)			1 (2.3%)		0 (0.0%)		
*ALL*	44 (84.6%)			7 (13.5%)		1 (1.9%)		

**Table 5:** Assessment and access quality of the public health services, n (%).

Access to SUS Services n (%)				
Access to SUS Services	Infrastructure	Health Professionals	Services	*p*-Value
Very good	7(77.8%)	2 (22.2%)	0 (0.0%)	0.88
Good	24 (75.0%)	6 (18.7%)	2 (6.3%)	
Neither good nor bad	62 (75.6%)	11 (13.4%)	9 (11.0%)	
Bad	33 (78.6%)	4 (9.5%)	5 (11.90%)	
Very bad	10 (76.9%)	1 (7.7%)	2 (15.4%)	
ALL	136 (76.4%)	24 (13.5%)	18 (10.1%)	

## Data Availability

The data presented in this study are available on request from the corresponding author. The data are not publicly available due to privacy and ethical restrictions.

## Supplementary Materials

The following supporting information can be downloaded at: https://www.mdpi.com/article/10.3390/ijerph21060721/s1, Survey Instrument S1.
