# Supplementary material for: Users’ Perceptions of Access to and Quality of Unified Health System Services in Brazil: A Cross-Sectional Study and Implications to Healthcare Management Challenges
Source: Int J Environ Res Public Health. 2024 May 31;21(6):721. doi: 10.3390/ijerph21060721 (PMC11204194; doi:10.3390/ijerph21060721)
Supplement: Supplementary file 1 [file ijerph-21-00721-s001.zip › ijerph-2967425-supplementary.pdf]

INSTRUMENT RESEARCH
SECTION A

Q01- REGION: 1. [ ] Metropolitan 4. [ ] North Fluminense 2.[ ] dos Lagos 3. [ ] Serrana

Q02- SEX:

1. [ ] Male
2. [ ] Female
3. [ ] Other
4. [ ] Don't know
5. [ ] Didn't answer

Q03- AGE (years): ___ years

Q04- MARITAL STATUS:

1. [ ] Single
2. [ ] Married
3. [ ]Widower
4. [ ] Stable relationship (friend)
5. [ ] Divorced or legally separated
6. [ ] Other
7. [ ] Don't know
8. [ ] No answer

Q05- SKIN COLOR:

1. [ ] White
2. [ ] Black
3. [ ] Brown (brunette, mulata, cabocla cafuza, mameluca, mixed race)
4. [ ] Indigenous
5. [ ] Quilombola
6. [ ] Yellow (Japanese, Chinese, Korean)
7. [ ] Other
8. [ ] Do not know
9. [ ] Did not answer

Q06- EDUCATION:

01. [ ] Illiterate
10. [ ] Incomplete elementary education (1st grade)
11. [ ] Complete elementary education 1st grade)
20. [ ] Incomplete high school education (2nd grade)
21. [ ]Complete secondary education (2nd grade)
30. [ ] Incomplete higher education (undergraduate)
31. [ ] Complete higher education (undergraduate)
40. [ ] Lato sensu postgraduate course (Specialization, MBA)
50. [ ] Master's
60.[ ] Doctorate
88. [ ] Don't know
99. [ ] No answer

Q07- TOTAL INCOME IN THE HOUSEHOLD: Adding the income of all the residents of your residence, what would be the total amount?

1. [ ] Up to 1 minimum wage (ÿR$ 1320.00)
2. [ ]1-2 minimum wages (R$ 1320.01 to R$ 2640.00)3. [ ] 2-3minimum wages (R$ 2640.01 to R$ 3960.00)

4. [ ] 3-5 minimum wages (R$ 3960.01 to R$ 6600.00)
5. [ ] 5-10 minimum wages(R$ 6600.01 to R $13200.00)
6. [ ] 10-20 minimum wages (R$ 13200.01 to R$26400.00)
7.[ ] More than 20 minimum wages (ÿR$ 26,400.01) _______
8.[ ] Don't now
9.9.[ ] Didn't answer

Q08- NUMBER OF PEOPLE IN THE HOUSEHOLD?

1. [ ] one
2. [ ] two
3. [ ] Three
4. [ ] Four or more

Q09- DO YOU HAVE A HEALTH PLAN? 1. [ ] Yes 2. [ ] No 8. [ ] I don't know 9. [ ] No response

Q10- DO YOU USE PUBLIC HEALTH SERVICES? 1. [ ] Yes 2. [ ] No 8. [ ] I don't know 9. [ ] No answer

SECTION B - CLINICAL CONDITIONS

Have you ever been told by a doctor or other healthcare professional that you have or have had the following

illnesses?Q101- Hypertension: 1.[ ] Yes 2. [ ] No 8. [ ] I don't know

Q102- Diabetes Mellitus: 1.[ ] Yes 2. [ ] No 8. [ ] I don't know

Q103- Heart diseases: 1.[ ] Yes 2. [ ] No 8. [ ] I don't know

Q104- Anxiety or depression: 1.[ ] Yes 2. [ ] No 8. [ ] I don't know

Q105- Arthritis, osteoarthritis, rheumatism: 1.[ ] Yes 2. [ ] No 8. [ ] I don't know

Q106- Stroke (cerebrovascularaccident or "stroke"): 1. [ ] Yes 2. [ ] No 8. [ ] I don't know

Q107- Dyslipidemia (high cholesterol and/or high triglycerides): 1. [ ] Yes 2. [ ] No 8. [ ] I don't know

Q108- Chronic lung disease (asthma, bronchitis, emphysema, other): 1. [ ] Yes 2. [ ] No 8. [ ] I don't know

Q109- Other illness(es) lasting MORE THAN SIX MONTHS: 1. [ ] Yes 2. [ ] No Which one(s)?__________

SECTION C - MEDICATION USE

Q201A- Do you use PRESCRIPTION medicines? 1. [ ] Yes 2. [ ] No 3. [ ] I don't know

Q201B- How many? 1. [ ] One 2. [ ] Two 3. [ ] Three 4. [ ] Four or more 88. [ ] I don't know 99. [ ] Not applicable

Q302A- Do you use medicines WITHOUT A PRESCRIPTION
? 1. [ ] Yes 2. [ ]No 3. [ ] I don't know

Q302B- How many? 1. [ ] One 2. [ ] Two 3. [ ] Three 4. [ ] Four or more 88. [ ] I don't know

99. [ ] Not applicable

Q302C- In which cases do you use any medicine WITHOUT A PRESCRIPTION? (You can select more than one option)

1. [ ] When you have medicine at home.
2. [ ] When you have taken this medicine before.
3.[ ] When you know someone who has already taken it and recommended it.
4. [ ] When you get the easy remedy.

5. When you receive a recommendation at the pharmacy. ________________________________

6. [ ] When you read the leaflet or other source of information(internet/social network).
7. [ ] Other:_______

8. [ ] I don't know

9. [ ] Not applicable

Q401- Do you trust the medicines you use?

1. [ ] Always 2. [ ] Most of the time 3. [ ] Rarely 4. [ ] Never 8. [ ] I don't't know

Q402- Do you receive medical advice regarding prescribed medications?

1. [ ] Always 2. [ ] Most of the time 3. [ ] Rarely 4. [ ] Never 8. [ ] I don't't know

Q403- How do you consider the medical advice you receive regarding prescribed medications:

1.[ ] Easy to understand

2. [ ] Confusing

3. [ ] Difficult

4. [ ] I don't receive guidance

8. [ ] I don't know

9. [ ] No response

Q404- Do you receive guidance from the pharmacist about the medicines do you use?

1. [ ] Always 2. [ ] Most of the time 3. [ ] Rarely 4. [ ] Never 8. [ ] I don't't know

Q405- How do you consider the pharmaceutical advice you receive about the medicines you use:

1. [ ] Easy to understand 2. [ ] Confusing 3. [ ] Difficult 4.[ ] I don't receive guidance 8. [ ] I don't know9.[ ] No answer

Q406- I will tell you some types of common difficulties that people face when dealing with medication.

Answer if you face each difficulty that I am going to talk about:

[Check all the options where the respondent says YES and asks "Any others?"]

1.[ ] Get the medicine.

2. [ ] Remember when to take your medicine

3. [ ] Using many medications a day.

4. [ ] Read what is written

5. [ ] Adapt the use of the medicine to your work schedule.

6. [ ] Confusing medicines that look on the packaging

7. [ ] Other:_______________________________________

8. [ ] Not using alcoholic beverages while taking any medication

0. [ ] None.

88. [ ] Don't know.

99. [ ] No response

SECTION D - PERCEPTIONS AND USE OF PUBLIC HEALTH SERVICES

Q501- Do you use public health services (SUS)?

1.[ ] Always 2.[ ] Often 3.[ ] Sometimes 4.[ ] Rarely 5.[ ] Never 8.[ ] I don't know 9.[ ] I didn't want to answer

Q502- What services do you use or have used from the SUS?

1.[ ] Medical consultation 2.[ ] Dental consultation 3.[ ] Vaccination 4.[ ] Surgery 5.[ ] Acquisition of Medication 6. [ ] Organ transplant 7.[ ] Various services 8.[ ] Prenatal /Preventive 9. [ ] None 10.[ ] Don't know 11.[ ] Didn't want toanswer

- If you answered that you NEVER USED (Q501) AND DID NOT USE ANY SUS SERVICE (Q502), thank you for participating and end the interview.

- **If you answered any of the other items in the previous questions, continue below.**

Q503- For you, the Unified Health System is:

1.[ ] Indispensable/Essential
2.[ ] Complement the private network
3. [ ] Indifferent
4 [ ] Unnecessary
8.[ ]] I don't know

9.[ ] I didn't want to answer

Q504- Do you know of any SUS program or initiative that is:

1.[ ] Yes 2.[ ] No 3.[ ] Don't know 4.[ ] Didn't want to answer

Q505- Are you registered with a Health Unit and/or Municipality's Family Health Strategy?

1.[ ] Yes 2.[ ] No 3.[ ] Don't know 4.[ ] Didn't want to answer

Q506- Do you collect medicines from a public health unit that is registered

1.[ ] Yes 2.[ ] I didn't want to answer 8.[ ] Don't know 9.[ ] No

Q507- What is your opinion about this type of service to be carried out by UBS and/or ESF?

1.[ ] Very good 2.[ ] Good 3.[ ] Neither good nor bad bad 4.[ ] Bad 5.[ ] Very bad8.[ ] I don't know
9.[ ] I didn't want to answer

Q508- Your medical appointments are carried out:

1.[ ] Only in the SUS 2.[ ] Only through the Health Plan 3.[ ] SUS and HealthPlan 8.[ ] Don't know 9.[ ] Didn't want toanswer

Q509- Do you consider the process for scheduling medical appointments with a general practitioner through theSUS:

1.[ ] Very good 2.[ ] Good 3.[ ] Neither good nor bad 4.[ ] Bad 5.[ ] Very bad8.[ ] I don't know 9.[ ] I didn't want to answer

Q510- Do you consider the process for scheduling appointments with specialists (ophthalmologist/ cardiologist)through the SUS:

1.[ ] Very good 2.[ ] Good 3.[ ] Neither good nor bad 4.[ ] Bad 5.[ ] Very bad8.[ ] I don't know 9.[ ] I didn't want to answer

Q511- Do you consider the medical care offered by the SUS to be:

1.[ ] Very good 2.[ ] Good 3.[ ] Neither good nor bad 4.[ ] Bad 5.[ ] Very bad8.[ ] I don't know 9.[ ] I didn't want to answer

Q512- Do you consider access to public health services as:

1.[ ] Very good 2.[ ] Good 3.[ ] Neither good nor bad 4.[ ] Bad 5.[ ] Very bad8.[ ] I don't know 9.[ ] I didn't want to answer

Q513- One of the objectives of our study is to evaluate citizens' perception of access to healthcare, which means "thecapacity of the healthcare system to provide the necessary care and services, at the right time and in the right place". ***How do you evaluate access to health services offered by the SUS:***

1.[ ] Very good 2.[ ] Good 3.[ ] Neither good nor bad 4.[ ] Bad 5.[ ] Very bad8.[ ] I don't know 9.[ ] I didn't want to answer

Q514- You were not served and/or were unable to access some type of service in the SUS in the last two years:

1.[ ] Yes 2.[ ] No 8.[ ] Don't know 9.[ ] Didn't want to answer If yes, what type of service?

Q515- For you, what is the most important thing associated with the quality of health services:

1.[ ] Agility in scheduling service 2.[ ] Geographical ease of location where the service will be performed 3. [ ] Agility in service. 4.[ ] Friendly welcome from professionals 4.[ ] Satisfactory environment (physical structure, comfort,

cleanliness, accessibility) 5.[ ] Humanized service 8.[ ] I don't know 9.[ ] I didn't want to answer

Q516- In your opinion, what is the most important factor to be improved regarding public health services:

1.[ ] Infrastructure 2.[ ] Qualification of professionals 3.[ ] Greater ease of access to medicines 4.[ ] Expansion of the number of health service units (UBS/ESF/ Hospitals) 5. [ ] Expansion of services offered (e.g. increase in the numberof consultations) 6. [ ] Easier access to medicines 8.[ ] I don't know 9.[ ] I didn't want to answer 10.[ ] Other:_________________

Q517- How do you evaluate the quality of health services offered by the SUS:

1.[ ] Very good 2.[ ] Good 3.[ ] Neither good nor bad 4.[ ] Bad 5.[ ] Very bad8.[ ] I don't know 9.[ ] I didn't want to answer

Q518- Have you ever had to use SUS services in another municipality other than yours?

1.[ ] Always 2.[ ] Often 3.[ ] Sometimes 4.[ ] Rarely 5.[ ] Never 6.[ ] I don't know 7.[ ] I didn't want to answer

Q519- If yes, which one? 1.[ ] Clinical consultation 2.[ ] Surgery 3.
[ ] Consultation with a specialist 4.[ ] I don't know 7.[ ] I didn't want to answer

Q520- How do you purchase your medicines?

1.[ ] Only privately (purchase in drugstores/ private pharmacies).2.[ ] Only free of charge in PUBLIC PHARMACIES
3.[ ] Both free of charge and privately.
4.[ ] Donation from friends/ family.8.[ ] I don't know
9.[ ] I didn't want to answer

Q521- Is this place far from your home? medicines provide information and/or guidance about

1. Yes [_] 2.More or less [_] 3.No [_] 8.I don't know [_]

Q522- How do you get to the place to purchase your medicine through SUS? *[You can select more than one option.]*

1.[ ] Walking 2.[ ] Bus and/or public transport 3.[ ] Car 4.[ ] Motorcycle 5.[ ]Bicycle 6.[ ] Other:____________

8.[ ] I don't know 9.[ ] I didn't want to answer

Q523- Do the employees of the SUS PUBLIC PHARMACIES where you collect your medicines serve you with respect andcourtesy?

1.[ ] Always 2.[ ] Often 3.[ ] Sometimes 4.[ ] Rarely 5.[ ] Never 8.[ ] NS 9.[ ] NR

Q524- Is the SUS PUBLIC PHARMACY where you get your medicines a clean and organized environment?

1.[ ] Always 2.[ ] Often 3.[ ] Sometimes 4.[ ] Rarely 5.[ ] Never 8.[ ] NS 9.[ ] NR

Q525- The opening hours of the SUS PUBLIC PHARMACY where you look for medicines are:

1.[ ] Very good 2.[ ] Good 3.[ ] Neither good nor bad 4.[ ] Bad 5.[ ] Very bad 8 .[ ] NS 9.[ ] NR

Q526- In the last three months, have you SEARCHED for any medicine in SUS PUBLIC PHARMACIES?

1.[ ] Always 2.[ ] Often 3.[ ] Sometimes 4.[ ] Rarely 5.[ ] Never 8.[ ] NS 9.[ ] NR

Q527- In the last three months, how often did you GET the medicines you were looking for FROM SUS PUBLICPHARMACIES?

1.[ ] Always 2.[ ] Often 3.[ ] Sometimes 4.[ ] Rarely 5.[ ] Never 8.[ ] NS 9.[ ] NR

Q528- Normally, how long do you wait to collect medicines from pharmacies?

1.[ ] The same day 2.[ ] The next day Three days later 4.[ ] One week later
5.[ ] 15 days later 6.[ ] More than 15 days later 7.[ ] Other:_____8.[ ] I don't know 9.[ ] NR

Q529- When you pick up medicine from PHARMACIES PUBLIC SUS, the employees who deliver the how to use them?

1.[ ] Always 2.[ ] Often 3.[ ] Sometimes 4.[ ] Rarely 5.[ ] Never 8.[ ] NS 9. [ ] NR

Q530- When you pick up medicines from public SUS pharmacies, do you receive guidance on how to store medicines at home?

1.[ ] Always 2.[ ] Often 3.[ ] Sometimes 4.[ ] Rarely 5.[ ] Never 8.[ ] NS 9.[ ] NR

Q531 - Is the pharmacist or other employee of SUS PUBLIC PHARMACIES available when you need to answerquestions about medicines?

1.[ ] Always 2.[ ] Often 3.[ ] Sometimes 4.[ ] Rarely 5.[ ] Never 8.[ ] I don't know 9.[ ] I didn't want to answer

Q532 - Have you already met the pharmaceutical professional at the public unit you frequent (ESF, UBS, medicationdispensing)?

1.[ ] Always 2.[ ] Often 3.[ ] Sometimes 4.[ ] Rarely 5.[ ] Never 8.[ ] I don't know 9.[ ] I didn't want to answer

Q533 - If yes, do you know what services are provided by the pharmaceutical professional?

1. Yes [_] 2.More or less [_] 3.No [_] 4.I don't know [_]

Q534 - In your municipality, is there a pharmacist in a Basic Health Unit and/or ESF?

1.[ ] Yes 2.[ ] No 8.[ ] I don't know 9.[ ] I didn't want to answer

Q535 - How do you evaluate the role of the pharmacist in the process of providing guidance on the use of medicines:

1.[ ] Indispensable/Essential 2.[ ] Indifferent 3.[ ] Unnecessary 8.[ ] I don't know/ I've never received advice from a pharmacist 9.[ ] I didn't want to answer

Q536 - In your opinion, what is the main contribution of the pharmacist to Primary Health Care?

1.[ ] Organization of administrative demands regarding medicines 2.[ ] Pharmaceutical guidance/care on the use of medicines 3.[ ] Contribute to the health actions and activities of a multidisciplinary team 8.[ ] I don't know 9.[ ] I didn't want to to respond

THANK YOU FOR YOUR PARTICIPATION AND AVAILABILITY!
